# White Matter Regions With Low Microstructure in Young Adults Spatially Coincide With White Matter Hyperintensities in Older Adults

**DOI:** 10.3389/fnagi.2019.00345

**Published:** 2019-12-10

**Authors:** Patrick J. Lao, Robert S. Vorburger, Atul Narkhede, Yunglin Gazes, Kay C. Igwe, Juliet Colón, Erica Amarante, Vanessa A. Guzman, Briana S. Last, Christian Habeck, Yaakov Stern, Adam M. Brickman

**Affiliations:** ^1^Taub Institute for Research on Alzheimer’s Disease and the Aging Brain, College of Physicians and Surgeons, Columbia University, New York, NY, United States; ^2^Institute of Applied Simulation, School of Life Sciences and Facility Management, Zurich University of Applied Sciences, Wädenswil, Switzerland; ^3^Department of Neurology, College of Physicians and Surgeons, Columbia University, New York, NY, United States

**Keywords:** white matter microstructure, white matter macrostructure, diffusion-weighted imaging, white matter hyperintensity, across the lifespan

## Abstract

Microstructural and macrostructural white matter damage occurs frequently with aging, is associated with negative health outcomes, and can be imaged non-invasively as fractional anisotropy (FA) and white matter hyperintensities (WMH), respectively. The extent to which diminished microstructure precedes or results from macrostructural white matter damage is poorly understood. This study evaluated the hypothesis that white matter areas with normatively lower microstructure in young adults are most susceptible to develop WMH in older adults. Forty-nine younger participants (age = 25.8 ± 2.8 years) underwent diffusion-weighted imaging (DWI), and 557 older participants (age = 73.9 ± 5.7 years) underwent DWI and T2-weighted magnetic resonance imaging (MRI). In older adults, WMH had a mostly periventricular distribution with higher frequency in frontal regions. We found lower FA in areas of frank WMH compared to normal-appearing white matter (NAWM) in older adults. Then, to determine if areas of normatively lower white matter microstructure spatially overlap with areas that frequently develop macrostructural damage in older age, we created a WMH frequency map in which each voxel represented the percentage of older adults with a WMH in that voxel. We found lower normative FA in young adults with regions frequently segmented as WMH in older adults. We conclude that low white matter microstructure is observed in areas of white matter macrostructural damage, but white matter microstructure is also normatively low (i.e., at ages 20–30) in regions with high WMH frequency, prior to white matter macrostructural damage.

## Introduction

White matter macrostructural damage due to small vessel disease, measured as white matter hyperintensity (WMH), has been linked to suboptimal cognitive outcomes (Gunning-Dixon and Raz, 2000; Gunning-Dixon et al., 2009), Alzheimer’s disease (AD) risk (Brickman et al., 2009; Brickman, 2013), emotional and motoric dysfunction (Murray et al., 2010), and risk of later development of stroke (Fazekas et al., 1993; Debette et al., 2010) in older adults. There has been recent interest in understanding the nature of the regional distribution of WMH (Yoshita et al., 2006; Brickman et al., 2012) and the extent to which WMH represent a “tip-of-the-iceberg” phenomenon, in which the hyperintense signal suggests widespread white matter microstructural damage (Zhan et al., 2009; Maillard et al., 2011, 2014; Promjunyakul et al., 2018). The most widely accepted biological model of WMH etiology is that damage to small blood vessels perfusing white matter leads to chronic hypoperfusion, ultimately damaging the tissue (Gurol et al., 2006). In the context of neurodegenerative diseases, other biological explanations, such as Wallerian-like degeneration, suggest that primary neuronal damage from neuropathology leads to “dieback” of connecting axons (Coleman, 2005; McAleese et al., 2017).

While clinical-pathological correlation studies show that radiologically defined WMH are strongly associated with macrostructural damage in white matter secondary to small vessel ischemic changes (Pantoni and Simoni, 2003; Jagust et al., 2008; Erten-Lyons et al., 2013), they also suggest widespread diminished microvascular density among individuals with marked WMH, even in white matter that is free of frank radiological abnormalities (Moody et al., 1995; Promjunyakul et al., 2018). More recently, the microstructure around tissue characterized as WMH has been interrogated with diffusion tensor imaging (DTI; Basser et al., 1994) to reveal subtle abnormalities in white matter surrounding WMH (Maillard et al., 2011, 2014; de Groot et al., 2013). Longitudinal analyses show relatively diminished markers of white matter microstructure (e.g., fractional anisotropy, FA) in normal-appearing white matter (NAWM) that subsequently develop WMH *de novo* (Maillard et al., 2011, 2014; de Groot et al., 2013; Brickman et al., 2015). These findings support a white matter “penumbra” (Maillard et al., 2011) in which microstructural disruption precedes the macrostructural damage represented by WMH, and also suggest that the underlying tissue damage extends beyond the borders of defined WMH.

Another distinct, but not mutually exclusive interpretation of the observation that white matter microstructure and WMH are related is that regional differences in normal white matter integrity in early life can render some areas more susceptible to white matter macrostructural damage in later life (Bartzokis, 2004; Yeatman et al., 2014). That is, in addition to local microstructural *damage* precipitating the formation of WMH, it is possible that white matter areas in the brain that show normatively lower microstructure, which can be characterized in healthy, young adults, may be most susceptible to developing WMH in later life.

The first aim of this study was to confirm that areas that are defined on T2-weighted fluid-attenuated inversion recovery (FLAIR) magnetic resonance imaging (MRI) as WMH have diminished white matter microstructure relative to NAWM in older adults. Then, by defining the normative white matter microstructure throughout the brain in young, healthy adults (i.e., well before any WMH are typically formed) and by defining regions that frequently develop WMH in late life, the second aim was to examine whether lower normative white matter microstructure confers susceptibility to future macrostructural changes.

## Methods

Participants were drawn from well-characterized young (*n* = 49, mean age ± standard deviation = 25.8 ± 2.8 years, 14 M/35F) and older (*n* = 557, 73.9 ± 5.7 years, 257 M/300F) samples. The older sample was drawn from a larger, ongoing, community-based study called the Washington Heights-Inwood Columbia Aging Project (WHICAP; Brickman et al., 2008). WHICAP is a community-based cohort designed to represent the demographic population surrounding the Columbia University Medical Center without the systematic exclusion of potential participants (Tang et al., 2001). Inclusion in WHICAP MRI sub-studies required that participants did not receive a diagnosis of dementia at the clinical follow-up visit preceding the scheduled MRI scan and that they did not have contraindications for MRI scanning (Brickman et al., 2008). Participants from WHICAP were included in the current analyses if they had undergone diffusion-weighted imaging (DWI), T1-weighted magnetization prepared rapid acquisition gradient echo (MPRAGE), or T2-weighted FLAIR imaging with a 3T MRI system, beginning in 2010. MRI data were acquired on a 3.0T Philips Achieva scanner for DWI (*b*-value = 800 s/mm^2^ along 16 gradient directions; field of view: 224 × 224 mm^2^; reconstruction matrix: 112 × 112; 81 slices; slice thickness: 2 mm; echo time (TE): 69 ms; TR: 8,185 ms), T1-weighted MPRAGE (field of view: 256 × 256 mm^2^, reconstruction matrix: 256 × 256; 165 slices; slice thickness: 1 mm; TE: 3 ms; repetition time (TR): 6.6 ms; inversion time (TI): 725 ms), and T2-weighted FLAIR (field of view: 240 × 240 mm^2^; reconstruction matrix: 560 × 560; 300 slices; slice thickness: 0.6 mm; effective TE: 338 ms; TR: 3,051 ms; TI: 50 ms; flip angle: 50°).

In the older sample, binary masks of WMH were derived as described previously (Brickman et al., 2011). Briefly, FLAIR images were intensity normalized and skull stripped/brain extracted with FMRIB Software Library (FSL; Jenkinson et al., 2012). For each FLAIR image, WMH were segmented by identifying voxels that were 1.2 standard deviations or greater than the mean intensity value of the image. T1-weighted images were co-registered to T2-weighted FLAIR images. T1-weighted images were segmented into tissue types (e.g., gray matter, white matter, cerebrospinal fluid) to obtain white matter masks in native space. All remaining voxels in the white matter (i.e., not labeled as WMH), were classified as NAWM. All segmented images were visually inspected for accuracy.

Parametric FA maps were computed in native MRI space after eddy current and susceptibility corrections using FSL. The average FA was computed over all voxels classified as WMH and as NAWM. This approach resulted in two samples for average FA values, one for WMH and one for NAWM, with a common sample size equal to the number of subjects. Distributions were checked for normality. To test the relationship between white matter microstructural damage in regions with macrostructural damage due to small vessel ischemia, we used a paired *t*-test for FA differences in WMH and NAWM among older adults. We also tested the association between FA within WMH and WMH volume among older adults.

We then computed a WMH frequency map from the older sample. The FLAIR images and binary WMH masks were spatially normalized to the T2 map in Montreal Neurological Institute (MNI)-defined standardized space (Evans et al., 2012), in the SPM12 toolbox using MATLAB2017a. The spatially normalized WMH masks were averaged to create a WMH frequency map (e.g., if 55 out of 557 subjects have a WMH in a given voxel, the frequency of a WMH in that voxel = 55/557 = 9.9%; Figure 2). Voxels in the WMH frequency map were binned based on the distribution of WMH frequency values which ranged from 0 to 22% and made into four separate masks representing voxels that were labeled WMH among 1%–5% (not including 0%, i.e., voxels in which no older participants demonstrated frank WMH), 6%–10%, 11%–15%, and 16%–20%. WMH frequency bins were chosen as four equally spaced intervals, representing easily interpretable ranges (i.e., 1%–5% would include a voxel that would be labeled hyper-intense in 1–5 individuals out of 100).

**Figure 1:**
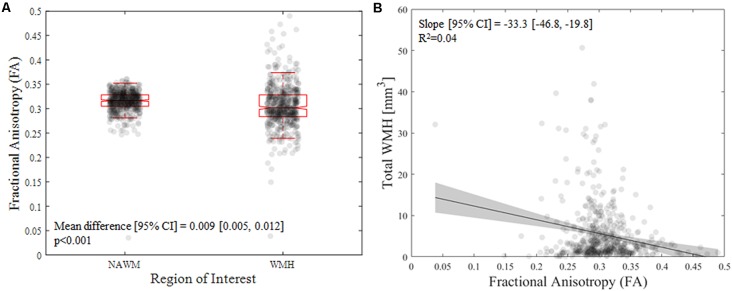
**(A)** Difference in mean fractional anisotropy (FA) value between nomal appearing white matter (NAWM) and white matter hyperintensities (WMH) in the older sample. **(B)** Association of mean FA value with WMH and the total WMH volume in the older sample.

**Figure 2:**
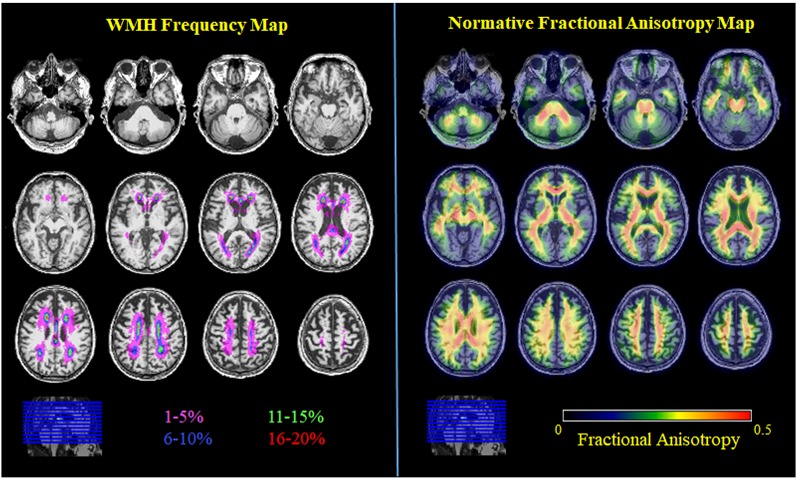
WMH frequency map (color coding represents WMH frequency bin) and normative FA map (average FA image of all young adults). All images are in MNI space.

The sample of young adults was a subset of a larger sample enrolled in an ongoing imaging study called the Reference Ability Neural Network study (RANN; Stern et al., 2014; Habeck et al., 2016). The inclusion criteria included native English speaking, right-handed, and at least a fourth-grade reading level. The exclusion criteria included myocardial infarction, congestive heart failure or any other heart disease, brain disorder such as stroke, tumor, infection, epilepsy, multiple sclerosis, degenerative diseases, head injury (loss of consciousness > 5 mins), mental retardation, seizure, Parkinson’s disease (PD), Huntington’s disease (HD), normal pressure hydrocephalus, essential/familial tremor, Down Syndrome, HIV Infection or AIDS diagnosis, learning disability/dyslexia, ADHD or ADD, uncontrolled hypertension, uncontrolled diabetes mellitus, uncontrolled thyroid or other endocrine disease, uncorrectable vision, color blindness, uncorrectable hearing and implant, pregnancy, lactating, any medication targeting central nervous system, cancer within last 5 years, renal insufficiency, untreated neurosyphilis, any alcohol and drug abuse within last 12 months, recent non-skin neoplastic disease or melanoma, active hepatic disease, insulin-dependent diabetes, history of psychosis or ECT, major depressive, bipolar, or anxiety disorder within the past 5 years), and MRI contraindications. Participants were included in the current analyses if they had undergone DWI at the time of analysis and were between the ages of 21 and 30. The goal was to derive normative white matter microstructure values in a sample of interest (e.g., young and healthy) to investigate deviations due to pathological conditions (e.g., aging and ischemic injury; Ziegler et al., 2014; Marquand et al., 2016). DWI data were acquired on a 3.0T Philips Achieva scanner (*b*-value = 800 s/mm^2^ along 56 gradient directions; field of view: 224 × 224 mm^2^; reconstruction matrix: 112 × 112; 75 slices; slice thickness: 2 mm; TE: 69 ms; TR: 7,645 ms) and processed with FSL to produce parametric FA maps for each subject in native space.

Similarly, parametric FA maps were computed for the young sample in native MRI space after eddy current and susceptibility corrections using FSL. The normative FA map was computed by averaging individual FA maps from young adults in standardized MNI space. To test for normative white matter microstructure differences in regions susceptible to developing future WMH, a multivariate ANOVA with unequal variance was used to test for differences in normative FA distribution, defined in the young sample, across WMH frequency bins, defined in the older sample. Specifically, an approximate Welch-Satterthwaite (W-S) correction on the degrees of freedom was used to account for the unequal variances across groups, which stems from the different noise quality of average FA in each WMH frequency bin [i.e., there are a different number of voxels in each WMH frequency bin (noise quality), but each subject contributes a mean FA value for each WMH frequency bin into the ANOVA (equal cell sizes)]. The W-S correction approaches the same result as an ANOVA when variances are equal (Satterthwaite, 1946).

Demographic differences between younger and older samples were tested with *t*-tests or Pearson’s chi-squared tests for continuous or categorical variables, respectively. While DWI sequences differed between cohorts, FA distributions were similar across cohorts and there were no direct comparisons between FA values in the young and older cohorts. Statistical tests were not adjusted for demographics or covariates because values were compared within subjects (i.e., FA in NAWM compared to WMH in each older participant; normative FA compared across WMH frequency bins in each younger participant).

## Results

By design, there was a difference in age [mean difference (95% confidence interval) = 48.3 (43.6, 49.9), *p* < 0.001]. There was also a difference in the proportion of women ($\chi_{\left(1\right)}^{2}$ = 4, *p* = 0.045), such that the young cohort had a higher proportion of women than the older cohort.

From the older cohort, across subjects, the presence of any WMH was 100% (18% had a total WMH volume below 1 cm^3^, 50% had a total WMH volume between 1 and 5 cm^3^, and 32% had a total WMH volume above 5 cm^3^). As expected, NAWM had higher FA compared with areas containing WMH in older adults [mean difference (95% confidence interval): 0.009 (0.005, 0.012), *p* < 0.001; Figure 1A]. Interestingly, higher WMH volume was associated with lower FA values within the WMH [*B* = −33.3 (−46.8, −19.8), *p* = 2E-6; Figure 1B].

**Figure 3:**
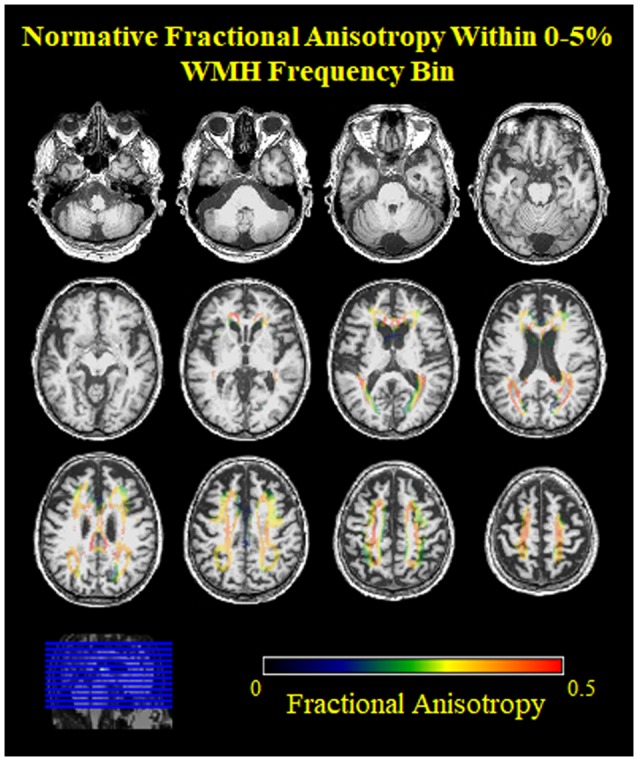
Normative FA values from young adults within the low frequency voxels for WMH in older adults. Note, the low FA (blue, green) values on the edges of the 0%–5% WMH bin, indicating inclusion of nearby gray matter.

Across voxels, the WMH frequency map (Figure 2) had a range of values from 0 to 22% with a mostly periventricular distribution and with higher WMH frequency in frontal regions. The number of voxels differed between each WMH frequency bin (1%–5%: 53,738 voxels, 6%–10%: 3,812, 11%–15%: 1,134, 16%–20%: 316). Using the John Hopkins University-International Consortium for Brain Mapping DTI 81 2 mm (JHU-ICBM-DTI81-2 mm) atlas to identify white matter tracts, the highest frequency voxels (16%–20% bin) fall within the genu of the corpus callosum and anterior and superior corona radiata. High-frequency voxels (11%–15%) fall within the genu of the corpus callosum, anterior and superior corona radiata, and posterior thalamic radiation. Intermediate frequency voxels (6%–10% bin) fall within the genu, body, and splenium of the corpus callosum, cingulum of the cingulate gyrus, superior longitudinal fasciculus, and tapetum, as well as the anterior, superior, and posterior corona radiata and posterior thalamic radiation. Low frequency voxels (1%–5% bin) fall within the genu, body, and splenium of the corpus callosum, anterior limb and retrolenticular part of the internal capsule, cingulum of the cingulate gyrus, superior longitudinal fasciculus, and tapetum, as well as the anterior, superior, and posterior corona radiata and posterior thalamic radiation. Notably, the low-frequency voxels (1%–5% bin) extend beyond the JHU-ICBM white matter atlas into the deep lobar gray matter of frontal, parietal, temporal, and occipital lobes (Admiraal-Behloul et al., 2004; Figure 3).

**Figure 4:**
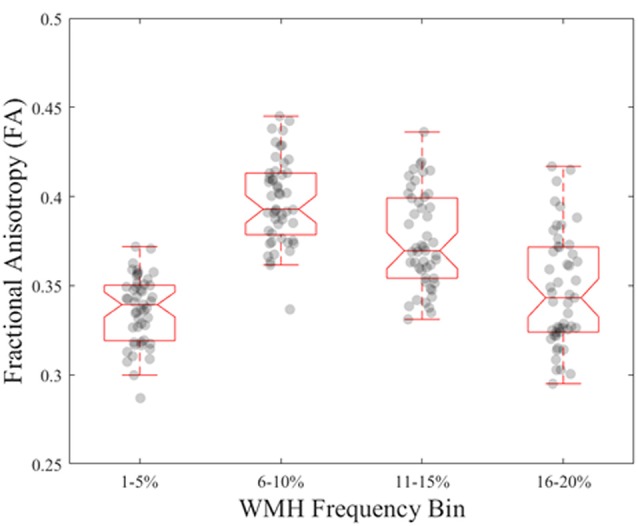
Normative FA values in young, healthy adults for each WMH frequency bin derived from older adults.

From the young cohort, the normative FA map (Figure 2) had a range of FA values from 0 to 0.7 with higher FA values in white matter tracts compared to gray matter, as expected. When comparing normative FA values in young adults as a function of WMH frequency derived from older adults, we found that FA values decreased with greater WMH frequency (mean ± standard deviation; 1%–5%: 0.33 ± 0.08; 6%–10%: 0.38 ± 0.06; 11%–15%: 0.37 ± 0.06; 16%–20%: 0.36 ± 0.06; *F*_(1.3,62)_ = 54.1, *p* < 0.001; Figure 4). The mean normative FA was low for the 1%–5% WMH frequency bin from the inclusion of gray matter beyond the JHU-ICBM atlas. While it was not strictly a monotonic decrease in normative FA across increasing WMH frequency, WMH frequency still explained approximately 53% of the variance in normative FA values in young adults.

## Discussion

We found a link between white matter microstructure and macrostructure that suggests both that there is microstructural damage in areas appearing radiologically as WMH and that low normative white matter microstructure in early life confers susceptibility to the development of future white matter macrostructural damage. Specifically, we confirmed that areas defined radiologically as WMH, presumably due to small vessel ischemia, in normal aging have reduced microstructure, indexed by lower FA values, compared with NAWM in older adults. We also find that white matter microstructure within WMH continues to decrease as the WMH progresses in older adults. We further demonstrated that white matter areas with normatively lower microstructure in young, healthy adults coincide with regions that are more likely to appear as WMH in older adults.

It should be noted that the purpose of the study was not to directly compare white matter microstructure in young and older adults but rather to use information from young, healthy adults to define a set of normative values for white matter microstructure and test whether white matter macrostructural damage in older adults varies as a function of its distribution. We observed an expected distribution of normative FA (white matter > gray matter; whole-brain range 0–0.7) in young, healthy adults. Separately, in our older cohort, we also observed a similar distribution of FA values (white matter > gray matter; whole-brain range 0–0.5). We also observed 32% of our older cohort to have a total WMH volume greater than 5 cm^3^ (Ramirez et al., 2016; prevalence of small vessel cerebrovascular disease = 16%–46% in elderly, Kapasi et al., 2017) and that the distribution of WMH frequency was predominantly in the frontal cortex (Figure 2), reflecting the anterior-posterior gradient typically observed in aging (Yeatman et al., 2014) rather than the posterior-predominant pattern typically observed in neurodegenerative diseases such as AD (Brickman et al., 2009; Lee et al., 2016).

This study joins previous efforts that have exploited the complementary information provided by DTI measures and T2-weighted MRI (Zhan et al., 2009; Brickman et al., 2015; Maniega et al., 2015; van Leijsen et al., 2018) in order to understand the relationship between microstructural and macrostructural white matter abnormalities in aging (Zhan et al., 2009; Croall et al., 2017; Promjunyakul et al., 2018). The implication of our findings is that regional developmental differences in white matter (e.g., amount of myelin, packing density, size of axons, etc.) could render some areas more vulnerable to ischemic injury in later life. It might be possible that areas of low white matter microstructure have fewer myelin-producing oligodendrocytes, making those regions more susceptible and less able to repair white matter areas exposed to chronic hypoperfusion (Bartzokis, 2004).

However, we observed an unexpectedly low FA in the lowest WMH frequency bin. This observation might be due to free water contamination, skewing the FA measurement to lower values (Fletcher et al., 2014). Most likely, this finding could be due to the fact that very low WMH frequency voxels only contain WMH in a small number of older participants by definition (e.g., a voxel with 1% WMH frequency means 1 out of every 100 older participants had a WMH in that voxel). These low-frequency voxels may be a result of imperfect WMH segmentation and/or spatial normalization, and may not represent an anatomical location that commonly develops WMH in later life (Figure 3). For example, voxels in the gray matter may be misclassified as a WMH or periventricular WMH may be over-warped into the ventricle. Both cases would lead to lower FA values in the lowest WMH frequency bin. A potential solution would be to use age-specific templates instead of the MNI template. While the overall contribution is the largest spatially, these events are rare (i.e., only need to occur in at least one scan).

Including deep lobar WMH may confound the results of a DTI-WMH analysis, not all WMH in gray matter are simply artifacts of improper segmentation or spatial normalization. Deep lobar WMH in gray matter are often segmented by various WMH segmentation algorithms, quantified in the four major lobes of the brain (Admiraal-Behloul et al., 2004), and can be considered small vessel cerebrovascular disease. As deep lobar gray matter is farther away from major blood vessels, it is perfused by small vessels extending from descending arteries. The brain regions supplied by small, terminal vessels are more susceptible to damage from chronic hypoperfusion. As such, excluding deep lobar WMH from the analysis would not necessarily be biologically meaningful and may only artificially improve results.

Major strengths of this study include the use of stringent inclusion/exclusion criteria to recruit a normative cohort of young, healthy adults (Stern et al., 2014) and two-cohort design, which allows us to infer some degree of causality and directionality. That is, in a young, healthy sample we have the ability to establish normative white matter microstructure patterns and then determine whether normative white matter microstructure differs spatially as a function of small vessel cerebrovascular disease in later life. If we addressed this question within the same cohort, we would not be able to disassociate whether white matter microstructure patterns were precipitating or resulting from small vessel cerebrovascular disease (or both). In other words, this approach has the potential to combine datasets with participants in restricted age ranges to dissociate early-life white matter microstructure from late-life pathology. Additionally, while former studies relied on two group classification analyses (i.e., WMH or NAWM), the present study used WMH frequency enabling a more detailed examination of the relationship between normative white matter microstructure and macrostructural damage.

There were also several limitations in this study. The lack of direct comparison between white matter microstructure in young and older samples obviates the need to harmonize MRI acquisition protocols across the two age groups, but we acknowledge that the different scanning protocols could bias our findings. Moreover, more advanced DWI acquisition sequences (e.g., diffusion kurtosis imaging), and/or an investigation of other DWI-derived metrics (e.g., mean diffusivity) would greatly benefit this type of analysis. Previous studies found that different metrics have different sensitivities for age-related changes in white matter microstructure (Hoagey et al., 2019; Slater et al., 2019). In addition to more advanced sequences, more advanced modeling of the free water component, particularly in the periventricular areas, would provide additional insight into this type of analysis because free water content may be a potential mediator of arterial stiffness and hemodynamics on white matter integrity (Fletcher et al., 2014; Maillard et al., 2017). A longitudinal study with both FA and WMH measures, including early life, is needed to confirm our findings. However, the longitudinal studies to date have been conducted among older adults only and show the future development of WMH in regions of low FA (de Groot et al., 2013), which could be interpreted as evidence that macrostructural damage emerges from microstructural damage, but not necessarily normatively low microstructure established during brain development in early adulthood. Recent studies showed that white matter may not reach peak maturation until midlife (Slater et al., 2019). A more in-depth study with various DWI-derived metrics and tract-specific analyses is a potential avenue for future work.

Our work suggests that regional differences in white matter maturation, as young as 20–30 years old, may lead to differential susceptibility to chronic hypoperfusion and contribute to future white matter macrostructural damage. Future work should focus on early life to identify causal factors in the mid- to late-life development of white matter macrostructural changes that have a substantial impact on cognition and the development of dementia due to AD.

## Data Availability Statement

The datasets generated for this study will not be made publicly available. Data related to this study can be requested at http://www.cumc.columbia.edu/adrc/investigators.

## Ethics Statement

The studies involving human participants were reviewed and approved by Columbia University Irving Medical Center IRB. The participants provided their written informed consent to participate in this study.

## Author Contributions

PL, RV, AN, KI, JC, EA, VG, and BL were primarily involved in data collection and pre-processing. PL and RV were involved in data analysis. PL, RV, and AB were primarily involved in manuscript writing. YG, CH, YS, and AB were primarily involved in the design of studies from which data was analyzed. All authors were involved in the manuscript preparation and editing.

## Conflict of Interest

The authors declare that the research was conducted in the absence of any commercial or financial relationships that could be construed as a potential conflict of interest.
